# Metformin mediates resensitivity to 5-fluorouracil in hepatocellular carcinoma *via* the suppression of YAP

**DOI:** 10.18632/oncotarget.10079

**Published:** 2016-06-15

**Authors:** Yu Tian, Bo Tang, Chengye Wang, Deguang Sun, Rixin Zhang, Nan Luo, Zhao Han, Rui Liang, Zhenming Gao, Liming Wang

**Affiliations:** ^1^ Division of Hepatobiliary and Pancreatic Surgery, Department of Surgery, The Second Affiliated Hospital of Dalian Medical University, Dalian, Liaoning, P.R. China; ^2^ Department of Hepatobiliary Surgery, Affiliated Hospital of Guilin Medical University, Guilin, Guangxi, P.R. China; ^3^ Department of Infection, The Second Affiliated Hospital of Dalian Medical University, Dalian, Liaoning, P.R. China

**Keywords:** metformin, hepatocellular carcinoma, 5-fluorouracil, YAP

## Abstract

Metformin plays an anti-proliferative role in tumor cells in many types of cancer. However, the correlation between metformin and sensitivity to chemotherapeutic agents in hepatocellular carcinoma (HCC) and the relevant mechanism are unclear. The present study showed that HCC patients with type 2 diabetes mellitus benefited from metformin administration, with a longer overall survival. Metformin resensitized Bel-7402/5-fluorouracil (Bel/Fu) cells to 5-fluorouracil (5-Fu) in vitro and in vivo, and the combination of metformin and 5-Fu inhibited cell proliferation, promoted cell apoptosis and induced G0/G1 cell cycle arrest in the Bel/Fu cells. Moreover, metformin repressed YAP by both decreasing the total protein expression and accelerating the phosphorylation of YAP. The inhibition of YAP subsequently promoted the expression of PTEN, and suppressed the Akt pathway. Therefore, the expression of P-gp and MRP1 was downregulated. Taken together, our findings suggested that metformin may increase sensitivity to chemotherapeutic agents by suppressing YAP in hepatocellular carcinoma.

## INTRODUCTION

Hepatocellular carcinoma (HCC) is of one the most aggressive malignancies worldwide. It is the sixth most common cancer on the global scale and the third-leading cause of cancer-related mortality [1, 2]. Surgical resection and liver transplantation may offer curative opportunities to select patients. However, the majority of HCC patients are diagnosed at an advanced stage and have lost the opportunity for surgical treatment [3, 4]. In this case, locoregional therapy, such as transarterial chemoembolization (TACE), is a feasible option, despite its low rate of efficacy and its associated small improvements in survival [5, 6]. One major drawback of TACE is the development of low sensitivity to chemotherapeutic agents [7]. Therefore, it is urgent to identify novel pharmacological strategies for HCC management.

Recent studies have shown that components of metabolic syndrome, including diabetes mellitus (DM), obesity and hyperlipidemia, are significant risk factors for HCC, and that the presence of DM is highly associated with the development of HCC [8, 9]. Metformin is a widely used oral anti-hyperglycemic agent for type 2 diabetes mellitus (T2DM). Systemically, it reduces glycemia by inhibiting hepatic gluconeogenesis and increasing insulin sensitivity in peripheral tissues. Recent epidemiological evidence has indicated that the use of metformin can decrease both the incidence and the mortality of multiple cancers [10], suggesting its potential role as an adjuvant agent for anti-cancer activity. However, the role and the underlying mechanism of metformin's effects on cancer-related chemotherapeutic agents' sensitivity remain unclear.

The Hippo signaling pathway is crucial in regulating tissue growth, stem cell activity and tumorigenesis [11]. YAP, first reported in 1994 as a binding partner of the SH3 domain of the Yes proto-oncogene product [12], is a key downstream effector of the Hippo signaling pathway. When the Hippo pathway is deactivated, YAP can translocate to the nucleus to activate gene expression by associating with a number of DNA-binding transcription factors, modulating diverse cellular functions, including proliferation, apoptosis, migration and differentiation [13, 14]. YAP is generally overexpressed in tumor tissues [15–17], and has been reported to modulate the epithelial-mesenchymal transition (EMT) and various pathways that are important for resistance to chemotherapy [18, 19]. Therefore, we hypothesized that YAP may play an important role in regulating chemotherapeutic sensitivity.

In this study, we showed that patients with HCC and T2DM who were treated with TACE benefited from metformin administration. Additionally, we presented evidence that metformin increased the sensitivity of Bel/Fu cells to 5-Fu both in vitro and in vivo, and that the suppression of YAP may be an important underlying mechanism of this effect.

## RESULTS

### Metformin administration resulted in longer OS in HCC patients with T2DM

To identify whether metformin facilitates resensitization to chemotherapeutic agents in HCC, we examined the curative effect of TACE in HCC patients with T2DM by administering metformin and other antidiabetic agents. The clinical features of the two studied patient cohorts, including age, gender, clinical stage, tumor size, tumor number and serum AFP level expression, are summarized in Table 1. No relationships were observed between the status of metformin administration and the other patient characteristics, including age, gender, serum AFP level, tumor size and tumor number.

**Table 1:** Association of metformin administration with patient's characteristics in hepatocellular carcinoma

Variables	Metformin	No Metformin	Total	*P*^a^
**Age (y)**				0.056
≤50	6 (35.3%)	11 (64.7%)	17	
>50	27 (58.7%)	19 (41.3%)	46	
**Sex**				0.847
Male	27 (51.9%)	25 (48.1%)	52	
Female	6 (54.5%)	5 (45.5%)	11	
**Tumor size (cm)**				0.425
≤5	24 (54.5%)	20 (45.5%)	44	
>5	9 (47.4%)	10 (52.6%)	19	
**AFP (ng/ml)**				0.961
≤20	20 (52.6%)	18 (47.4%)	38	
>20	13 (52%)	12 (48%)	25	
**Tumor number**				0.217
Solitary	30 (55.6%)	24 (44.4%)	54	
Multiple	3 (33.3%)	6 (66.7%)	9	

a χ^2^ test.

b median age.

**Figure 1:**
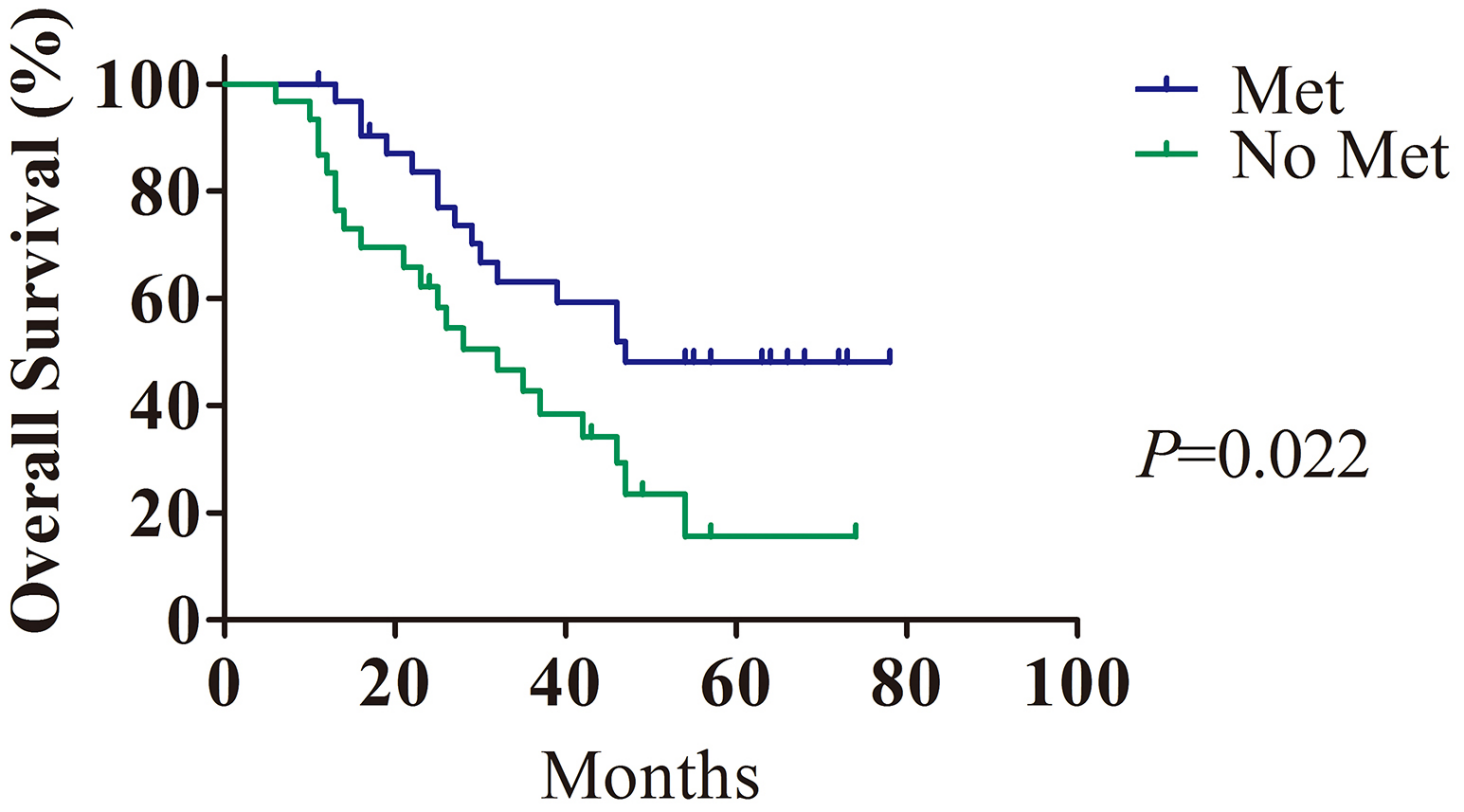
The prognostic value of metformin in HCC patients with T2DM Treatment with metformin in HCC patients with T2DM (n=33) was closely correlated with longer overall survival compared with treatment with other antidiabetic agents (n=30).

The median follow-up period was 35.1 months (ranging from 6 to 78 months) for the HCC patients. The difference in survival rate between the metformin group and the other antidiabetic agents group was evaluated. Kaplan-Meier analysis showed that metformin administration strongly predicted longer OS (*P*<0.01, Figure 1).

### Metformin increased sensitivity to chemotherapeutic agents of HCC in vitro and in vivo

**Figure 2:**
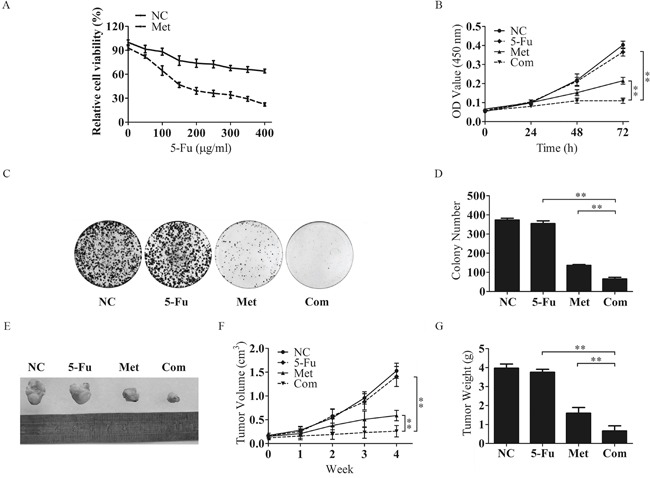
Metformin inhibits the proliferative capacity of Bel/Fu cells in combination with 5-Fu **A.** The sensitivity of Bel/Fu cells to 5-Fu was measured by CCK-8 assay. **B.** Bel/Fu cells were treated with different agents, and relative cell viability was measured by CCK-8 assay at 0 h, 24 h, 48 h and 72 h. **C** and **D.** Bel/Fu cells were plated in 6 cm dishes at a density of 1000 cells per dish. After 2 weeks of different treatments, the number of colonies was counted. **E.** Combined treatment with 5-Fu and metformin decreased tumor size. The representative images of Bel/Fu xenograft tumors injected subcutaneously and treated with NC, 5-Fu, Met and Com. **F.** The growth curve of tumors formed by Bel/Fu cells after treatment with NC, 5-Fu, Met and Com. **G.** The weights of tumors formed by Bel/Fu cells after different treatments at harvest time. **P < 0.01.

As metformin administration helps HCC patients with T2DM obtain a better prognosis, we performed an in vitro investigation to test whether metformin could enhance chemotherapeutic sensitivity. Bel/Fu cells treated with metformin exhibited significantly promotion of sensitivity to 5-Fu (Figure 2A). The same effect of metformin could be observed in HepG2 and SK-Hep-1 cells. Combination with metformin did enhanced efficiency of chemotherapeutic agents, such as 5-Fu, doxorubicin (DOX) and sorafenib (Sor) (Supplementary Figure S1A - S1H). Then, Bel/Fu cells were separately treated with negative control (NC), 100 μg/ml 5-Fu (5-Fu), 10 mM metformin (Met) and a combination of these agents (Com). Cell proliferation was evaluated by CCK-8 assay. The course of proliferation was assessed for 3 days after treatment. Met and Com treatments exhibited effective inhibition of cell proliferation compared with the 5-Fu group, especially in the Com group (Figure 2B). We further conducted cell colony formation assays to demonstrate whether metformin combined with 5-Fu inhibited cell proliferation in Bel/Fu cells. The number of colonies formed was 373.0 ± 9.8 for the NC cells, 354.3 ± 14.7 for the 5-Fu group, 136.3 ± 4.2 for the Met group and 65.0 ±8.9 for the Com group (Figure 2C and 2D).

To further examine the role of Met in regulating sensitivity to 5-Fu in vivo, we subcutaneously injected Bel/Fu cells into nude mice and treated them with the above agents. As expected, tumors grew more slowly in combined treatment than in single-agent treatment (Figure 2E-2G). These results indicated that metformin could resensitize Bel/Fu cells to 5-Fu.

### Combined treatment with 5-Fu and metformin promoted apoptosis in Bel/Fu cells

**Figure 3:**
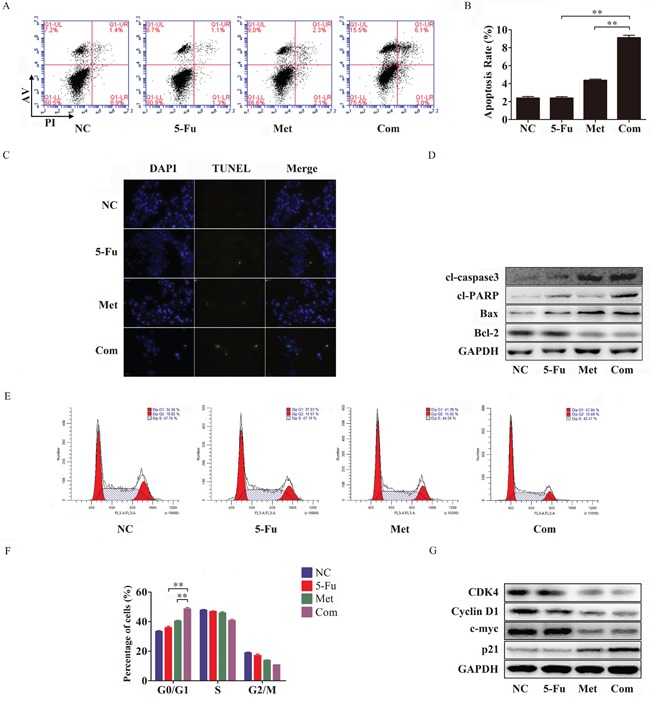
Metformin promotes apoptosis and arrests cell cycle progression in combination with 5-Fu in Bel/Fu cells **A** and **B.** Bel/Fu cells were pretreated NC, 5-Fu, Met and Com separately. After 48 h of treatment, flow cytometry was performed to analyze the rate of cell apoptosis. **C.** TUNEL assay was used to detect DNA damage after the different treatments. **D.** The protein levels of cl-caspase3, cl-PARP, Bax, and Bcl-2 were detected by western blotting. **E** and **F.** 24 h after different treatments, flow cytometry was performed to analyze the progression of the cell cycle. **G.** The protein levels of CDK4, cyclin D1, c-myc and p21 were detected by western blotting. **P < 0.01.

To determine whether metformin-induced increased sensitivity to 5-Fu in Bel/Fu cells was accompanied by apoptosis, the percentage of apoptosis was examined using flow cytometry after 48 h of incubation with the different agents. The percentage of apoptotic cells was significantly increased after combination therapy compared with single-agent therapy and negative control treatment. The percentage of apoptotic cells was 2.5 ± 0.1% in the NC group, 2.3 ± 0.2% in the 5-Fu group, 4.5 ± 0.1% in the Met group and 9.1 ± 0.5% in the Com group (Figure 3A and 3B).

The TUNEL assay is commonly used to detect fragmented DNA in cells that undergo apoptosis. In our study, Bel/Fu cells were pretreated with different agents for 48 h for TUNEL assay, and the cells were stained by TUNEL and DAPI and analyzed by fluorescence microscopy. As shown in Figure 3C, pretreatment with the combination of 5-Fu and metformin produced more green light than pretreatment with other agents, which suggested that combination treatment increased Bel/Fu cell apoptosis.

Subsequently, the up-regulation of the pro-apoptotic proteins cl-caspase3, cl-PARP and Bax and the down-regulation of the apoptosis suppressor Bcl-2 were observed in the combination treatment group compared with the other groups (Figure 3D).

### Combined treatment with 5-Fu and metformin arrested the cell cycle at G0/G1 in Bel/Fu cells

To explore whether metformin could resensitize Bel/Fu cells to 5-Fu by modifying the cell cycle, we examined cell cycle progression by flow cytometry. 5-Fu combined with metformin arrested the cell cycle at G0/G1 in Bel/Fu cells compared with the other groups (Figure 3E and 3F). 24 h after treatment with the different agents mentioned above, the percentage of cells in the G0/G1 phase was 33.4 ± 0.9% for the NC group, 35.8 ± 1.8% for the 5-Fu group, 40.4 ± 1.0% for the Met group and 48.6 ± 1.7% for the Com group.

Dysfunction of cell cycle checkpoint proteins can alter the progression of the cell cycle. We also examined the G1/S phase proteins cyclin D1, CDK4 and p21. Cyclin D1 and CDK4, which are responsible for the transition from G0/G1 phase to S phase, were downregulated following combined treatment. Additionally, p21, a cyclin-dependent kinase (CDK) inhibitor, was obviously induced following combined treatment (Figure 3G). Thus, these results demonstrated that the combined treatment group arrested Bel/Fu cells in the G0/G1 phase.

### Silencing YAP increased sensitivity to 5-Fu in Bel/Fu cells

**Figure 4:**
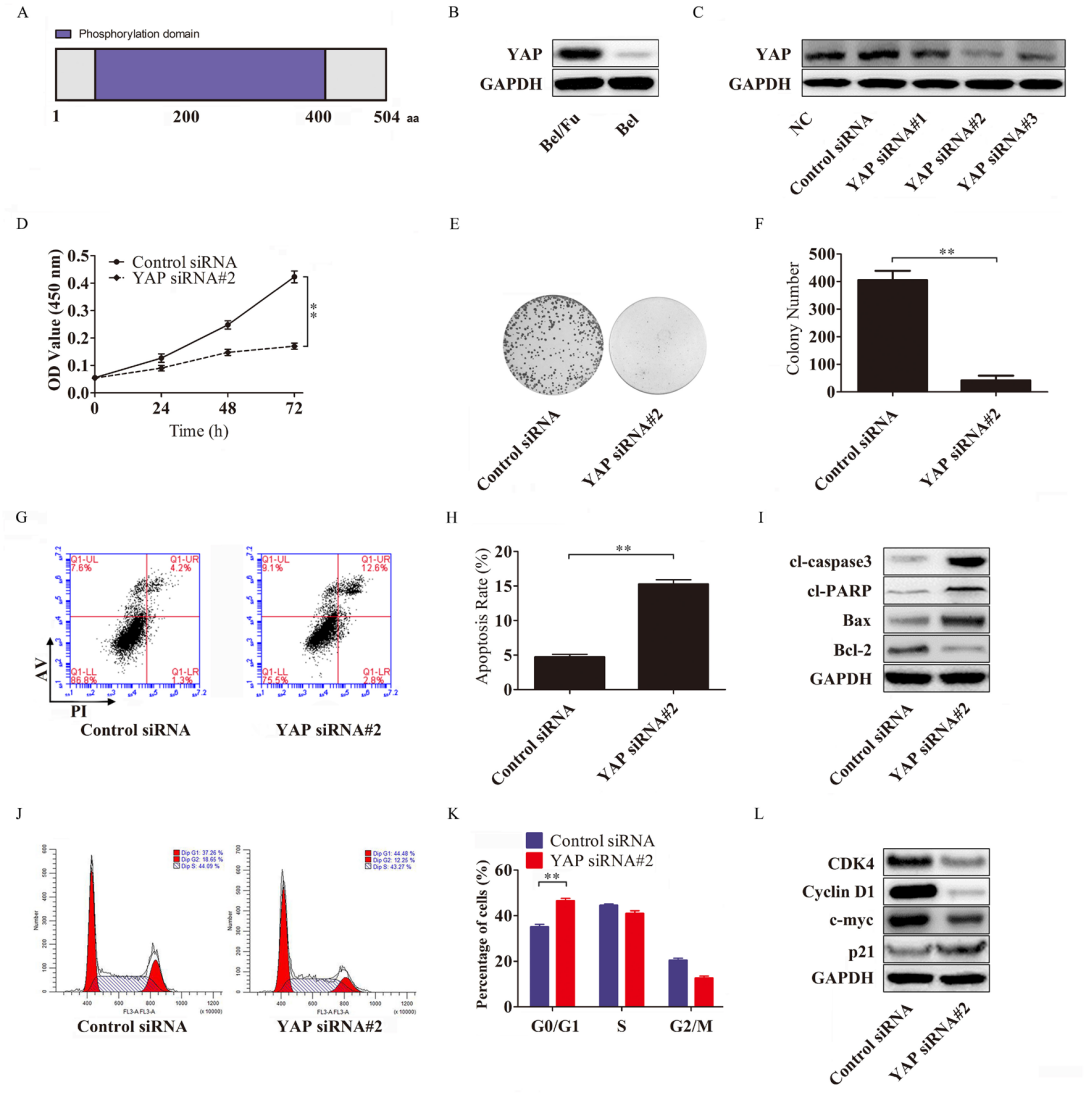
YAP knockdown resensitizes Bel/Fu cells to 5-Fu in Bel/Fu cells **A.** A schematic illustration of the human YAP protein. YAP consists of 504 amino acid residues and has 22 phosphorylation sites. **B.** The expression of YAP in Bel/Fu and Bel cells. **C.** Bel/Fu cells were transfected with siRNAs, and the expression of YAP was detected by western blotting. **D.** After the cells were transfected with control siRNA and YAP siRNA#2, relative cell viability was measured by CCK-8 at different time points. **E** and **F.** After being transfected with control siRNA and YAP siRNA#2, the cells were cultured in 100 μg/ml 5-Fu medium for 2 weeks, and colony numbers were counted. **G** and **H.** Flow cytometry detected the rate of apoptosis in different siRNA-transfected cells. **I.** Detection of apoptosis-related proteins. **J** and **K.** Flow cytometry detected cell cycle progression in different siRNA-transfected cells 24 h after siRNA transfection. **L.** Detection of cell cycle checkpoint proteins. **P < 0.01.

It has been reported that YAP, a protein abundant with phosphorylation sites (Figure 4A), plays an important role in cancer cell activity, especially in tumor-associated chemoresistance [20]. To investigate the function of YAP in regulating sensitivity to 5-Fu in HCC, we evaluated the expression of YAP in Bel/Fu and Bel cells. As shown in Figure 4B, the YAP protein was highly expressed in Bel/Fu cells.

To determine whether knockdown of YAP could increase the sensitivity of Bel/Fu cells to 5-Fu, YAP siRNAs were used to silence the expression of YAP. YAP siRNA#2 repressed YAP protein expression more effectively than other YAP siRNAs (Figure 4C), and the Bel/Fu cells transfected with YAP siRNA#2 displayed a lower proliferative capacity (Figure 4D), a decreased number of colonies (Figure 4E and 4F), an increased cell apoptotic rate (Figure 4G and 4H) and a larger number of cells arrested in the G0/G1 phase (Figure 4J and 4K) under 100 μg/ml 5-Fu than the control siRNA-transfected Bel/Fu cells.

Furthermore, the activation of the pro-apoptotic cl-caspase3, cl-PARP and Bax and the downregulation of the apoptosis suppressor Bcl-2 were associated with YAP silencing-induced apoptosis (Figure 4I). Cyclin D1 and CDK4, which are responsible for the transition from the G0/G1 phase to the S phase, were downregulated following YAP knockdown (Figure 4L).

These results indicated that the knockdown of YAP increased sensitivity to 5-Fu in Bel/Fu cells.

### Silencing YAP mediates P-gp and MRP1 downregulation by inhibiting the phosphorylation of Akt

**Figure 5:**
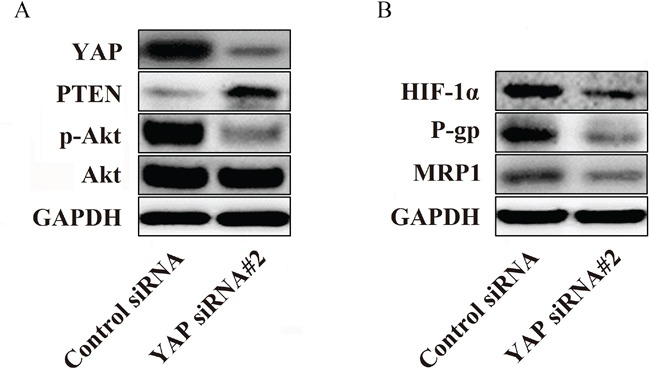
YAP regulates P-gp and MRP1 by regulating the phosphorylation of Akt in Bel/Fu cells **A.** The knockdown of YAP increased the expression of PTEN and decreased the phosphorylation of Akt in Bel/Fu cells. **B.** The suppression of HIF-1α, P-gp and MRP1 was observed after YAP knockdown.

Activation of Akt has been described in a variety of cancers, and the PTEN/Akt pathway plays a critical role in controlling the balance between pro- and anti-oncogenic pathways [21]. The aberrant expression of these proteins is always observed in cells that are not sensitive to chemotherapeutic agents [22]. Therefore, we examined the influence of YAP knockdown on PTEN, p-Akt and Akt protein levels by western blotting. As shown in Figure 5A, compared with the control cells, the knockdown of YAP in the Bel/Fu cells resulted in significant increases in PTEN, whereas the expression of p-Akt decreased remarkably but did not influence total Akt levels.

Hypoxia-inducible factor-1α (HIF-1α) is a downstream protein of Akt that transcriptionally regulates the expression of (P-glycoprotein) P-gp and multidrug resistance-associated protein 1, MRP1, which contribute to the development of chemoresistance [23]. Therefore, we investigated whether YAP could influence the expression of HIF-1α, P-gp and MRP1. Interestingly, western blotting illustrated that reduced expression levels of HIF-1α, P-gp and MRP1 were detected in YAP siRNA#2-transfected cells compared with control siRNA-transfected cells (Figure 5B). Therefore, these data may indicate a potential mechanism by which YAP regulates sensitivity to 5-Fu in Bel/Fu cells.

### Metformin downregulated expression of YAP and promoted YAP by activating AMPK

**Figure 6:**
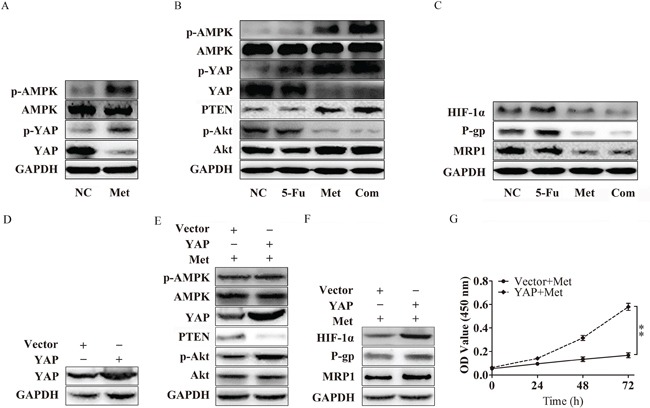
Metformin inhibits P-gp and MRP1 by suppressing YAP **A.** Bel/Fu cells pretreated with metformin activated AMPK, decreased the expression of YAP, and inhibited the phosphorylation of YAP. **B.** Bel/Fu cells were treated with NC, 5-Fu, Met, and the Com, both PTEN protein levels and the levels of phosphorylated AMPK, YAP and Akt were examined using western blotting. **C.** HIF-1α, P-gp and MRP1 decreased in the Met group and the Com group compared with the NC group and the 5-Fu group. **D.** The expression of YAP in Bel/Fu-vector and Bel/Fu-YAP cells. **E.** After treated with metformin, Bel/Fu-YAP cells showed lower expression of PTEN and higher expression of p-Akt than Bel/Fu-vector cells. **F.** After treated with metformin, Bel/Fu-YAP cells showed higher expression of HIF-1α, P-gp and MRP1 than Bel/Fu-vector cells. **G.** CCK-8 assay for proliferation analysis between Bel/Fu-vector and Bel/Fu-YAP cells after metformin treatment. **P < 0.01.

It has recently been reported that AMPK activation can induce the phosphorylation of YAP, which is important in regulating cytokine transcription in the nucleus [24]. In our previous work, we confirmed that metformin is an AMPK activator. We further evaluated whether metformin could influence the phosphorylation of YAP. As expected, increased phosphorylation of YAP was observed in the Met and Com groups compared with the 5-Fu and the NC group, in agreement with the level of phosphorylation of AMPK. Moreover, we also discovered that the expression of YAP also decreased with metformin treatment (Figure 6A). These results indicated that activation of AMPK by metformin may suppress YAP with respect to the levels of total protein and phosphorylated protein.

### Metformin inhibited P-gp and MRP1 via the suppression of YAP

In our study, we found that, in agreement with the previous presentation, metformin and combined treatment induced the phosphorylation of AMPK via the suppression of YAP. PTEN was significantly upregulated in the Met group and Com group, followed by the decreased phosphorylation of Akt (Figure 6B). Compared with the NC group and the 5-Fu group, the expression of HIF-1α, P-gp and MRP1 was obviously repressed (Figure 6C).

To verify whether YAP is a key regulator in metformin induced resensitivity to 5-Fu in Bel/Fu cells. We established YAP stable expression Bel/Fu cells. Western blotting showed YAP was overexpressed in Bel/Fu-YAP than Bel/Fu-vector (Figure 6D). After treatment with 10mM metformin, Bel/Fu-YAP showed a lower expression of PTEN, and higher expression of p-Akt than Bel/Fu-vector (Figure 6E). In addition, HIF-1α, P-gp and MRP1 expressed higher in Bel/Fu-YAP cells (Figure 6F). CCK-8 analysis showed overexpression of YAP blocked metformin induced resensitivity to 5-Fu (Figure 6G). These results further demonstrated that metformin may enhance chemotherapeutic sensitivity through the suppression of YAP.

## DISCUSSION

The low chemosensitivity of cancer cells has been implicated as a major challenge in anti-cancer treatment. Many factors have been identified in the development of insensitivity to chemotherapeutic agents: elevated expression of drug efflux transporters, changes in drug kinetics, amplification of drug targets or tumor heterogeneity, which comprise genetic variation, and the tumor microenvironment [25]. Ineffectiveness of chemotherapeutic agents in patients with advanced HCC has garnered the attention of increasing numbers of researchers.

Metformin, a biguanide derivative that is used widely to treat T2DM, reduces basal glucose output by suppressing gluconeogenesis and glycogenolysis in the liver and increasing glucose uptake by muscle. The mechanism involved in the effect of metformin is the liver kinase B1 (LKB1)-dependent activation of AMPK [26, 27]. Many reports have demonstrated that there is an association between T2DM and various types of cancer [28]. In addition, the combination of metformin with conventional chemotherapeutic agents could also enhance chemosensitivity [29, 30], with little research focusing on HCC. In this study, we evaluated the efficiency of metformin and other antidiabetic agents in TACE treated HCC patients with T2DM. Similar to the findings of previous studies, metformin administration was associated with longer overall survival.

Although much progress has been made regarding the role of the Hippo pathway in tumorigenesis and stem cell renewal and differentiation [12, 31], its function with respect to the response to chemotherapeutic agents is largely unknown. YAP, one of the most critical downstream effectors of the Hippo pathway, has been reported to form a complex with sequence-specific DNA-binding proteins and promotes the expression of genes that drive cell proliferation and inhibit apoptosis [15, 17]. Phosphorylation of YAP deactivated YAP and translocated it from the nucleus to the cytoplasm, which abolished its transcriptional activity. Overexpression of YAP is associated with chemoresistance in esophageal cancer, and its interaction with the TEAD binding site in the EGFR promoter resulted in increased EGFR expression at the transcriptional level, causing resistance to 5-Fu and docetaxel [32]. Dephosphorylation of YAP at Ser 127 dissociated it from 14-3-3 binding, leading to its nuclear assembly and transcriptional activation [33]. To explore the biological function of YAP in HCC cells, YAP-specific siRNA was transfected into Bel/Fu cells. YAP knockdown suppressed cell growth, promoted cell apoptosis and induced G0/G1 cell cycle arrest in Bel/Fu cells treated with 5-Fu. Interestingly, Bel/Fu cells treated with metformin also showed suppression of YAP, which demonstrated that YAP is a crucial molecule in metformin induced 5-Fu resensitivity.

AMPK, a cellular energy stress sensor activated by increasing AMP levels, can modulate cellular metabolism and function to coordinate cell growth with available energy [34]. It has been reported that AMPK can inhibit YAP in a Lats-dependent and Lats-independent manner. AMPK phosphorylates and stabilizes angiomotin (AMOTL1) to stimulate Lats activity, thus phosphorylating YAP on five serine residues, including Ser 127 [35]. AMPK mediates the YAP phosphorylation, especially on Ser 94, plays a critical role in YAP inhibition by disrupting the formation of the YAP-TEAD complex [36]. Consistent with previous studies, metformin induced AMPK activation, phosphorylated YAP and decreased the expression of YAP, leading to the further inhibition of YAP in Bel/Fu cells.

**Figure 7:**
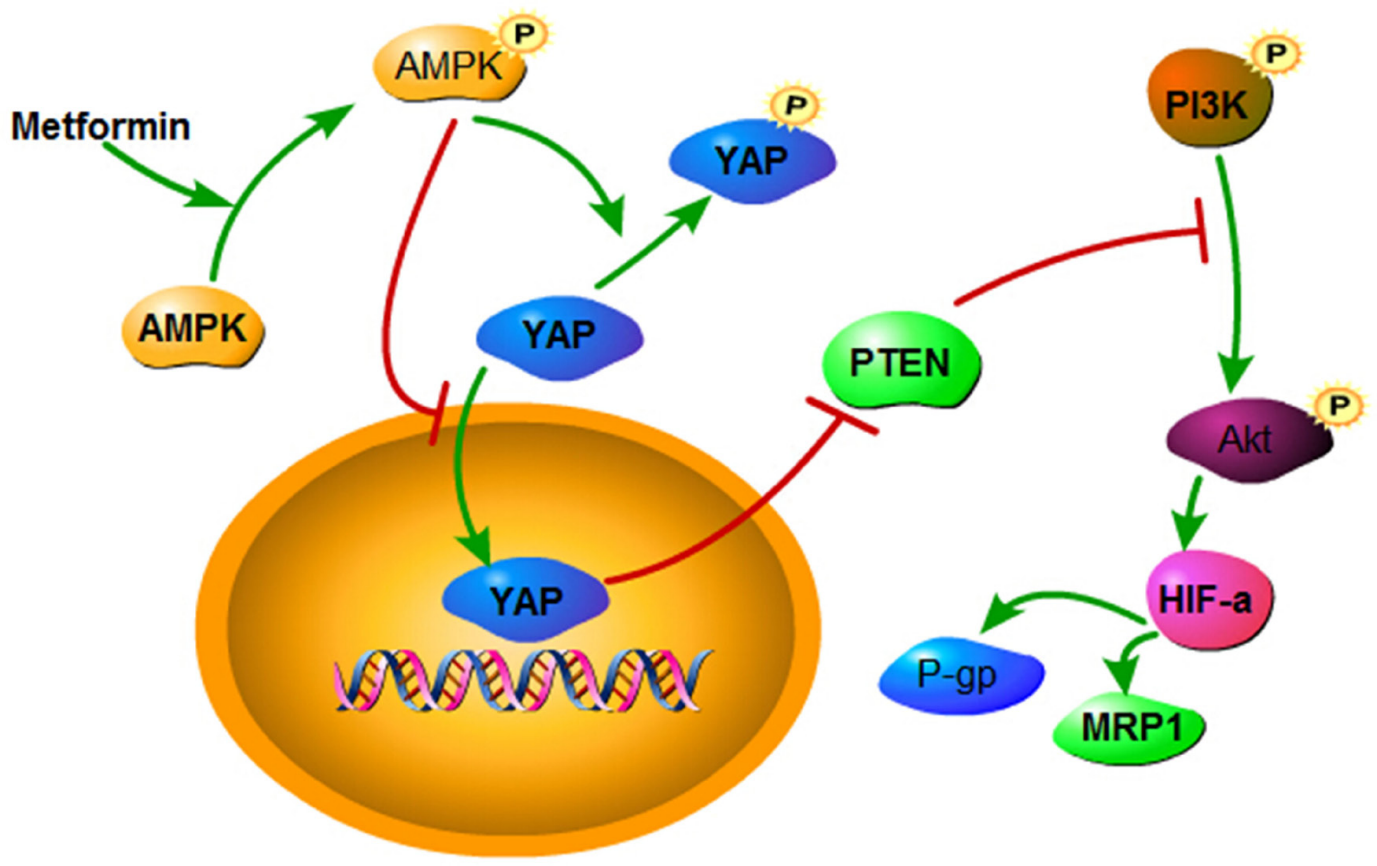
A schematic model elucidating the role of metformin in regulating the chemosensitivity of Bel/Fu cells

HIF-1α has been recognized as a mediator of the development of chemoresistance. As the key stimulator of HIF-1α, oxygen deprivation led to DDP and doxorubicin resistance in lung cancer cells [37]. P-gp and MDR1 are members of the drug transporter family, which are energy-dependent drug efflux pumps important in drug resistance [38]. They can be regulated by diverse signaling pathways, including the PI3K/Akt/HIF-1α pathway [39]. Kunliang Guan et al provided evidences that YAP mediates the major effects of the Hippo pathway by regulating gene expression, and among the YAP target genes is the miR-29 family, which inhibits PTEN, a tumor suppressor that inhibits the phosphorylation of Akt by targeting its 3′UTR [40]. In the current work, we silenced YAP and treated Bel/Fu cells with metformin. Both managements led to the upregulation of PTEN, following downstream HIF-1α downregulation, and reduced the expression of P-gp and MRP1. Moreover, overexpression of YAP suprressed Akt activation, and blocked inhibition of P-gp and MRP1 induced by metformin. Therefore, we suggest that the downregulation of P-gp and MRP1 by metformin in Bel/Fu cells may be caused by the suppression of YAP/PTEN/Akt (Figure 7).

In conclusion, our findings revealed that HCC patients suffering from T2DM benefited from metformin administration, and the combination with metformin enhanced the sensitivity of Bel/Fu cells to 5-Fu both in vitro and in vivo. This study illustrates a novel mechanism by which metformin can be used as a prospective chemotherapeutic agent or chemosensitizer in HCC treatment.

## MATERIALS AND METHODS

### Patients

A total of 63 HCC patients with type 2 diabetes mellitus (T2DM) who were diagnosed between March 2009 and December 2015 at the Second Affiliated Hospital of Dalian Medical University were retrospectively analyzed. All HCC patients received TACE treatment. The present study was approved by the Ethics Committees of the Second Affiliated Hospital of Dalian Medical University in accordance with the ethical guidelines of the Declaration of Helsinki. Consent was obtained from either the patient or the patient's family.

### Reagents and antibodies

Metformin and 5-Fu were obtained from Sigma-Aldrich (St. Louis, MO, USA). The Cell Cycle Detection Kit, Cell Counting Kit-8, Annexin V-fluorescein isothiocyanate (FITC) Apoptosis Detection Kit, TUNEL Apoptosis Detection Kit and BCA protein assay kit were purchased from KeyGen Biotech Co., Ltd (Nanjing, China).

Antibodies against caspase3, cleaved-caspase3 (cl-caspase3), PARP, cleaved-PARP (cl-PARP), Bax, Bcl-2, p-YAP, YAP, p-AMPK, AMPK, Akt, p-Akt, PTEN, HIF-1a, p21, c-myc and GAPDH were obtained from Santa Cruz Biotechnology, Inc. (Santa Cruz, CA, USA), the P-gp and MRP1 antibodies were obtained from Thermo Fisher Scientific Inc. (Rockford, IL, USA), and the cyclin D1 antibody was purchased from Abgent (San Diego, CA, USA).

### Cell culture and transfection

The human hepatocellular carcinoma cell lines, Bel-7402 (Bel) and Bel-7402/5-fluorouracil (Bel/Fu), were purchased from KeyGen Biotech Co., Ltd. HepG2, SK-Hep-1 were obtained from obtained from the tumor cell bank of the Chinese Academy of Medical Science. The cells were cultured in RPMI-1640 medium (Gibco-BRL, Carlsbad, CA, USA) supplemented with 10% FBS (Gibco-BRL, Carlsbad, CA, USA), 100 μg/ml penicillin and 100 μg/ml streptomycin at 37°C in a 5% CO_2_ atmosphere. The Bel/Fu cells were maintained in medium containing 20 μg/ml 5-Fu. YAP specific siRNAs and full-length YAP complementary DNA were prepared and transfected as described previously [19, 41].

### Cell viability assay

Approximately 5×10^3^ cells/well were seeded into the wells of 96-well plates. After being cultured for 24 h, the cells were treated with different agents or siRNA transfection. The cells were incubated at 37°C for another 24, 48 or 72 h, followed by exchanging the medium containing 100 μl of RPMI-1640 medium and 10 μl of CCK-8 reagent for 2 h at 37°C. Finally, light absorbance was measured at 450 nm using a microplate reader.

### Colony formation assay

Approximately 1000 cells/well were seeded in triplicate into 6-well plates. Then, 24 h after seeding, the cells were treated with different agents or siRNA transfection. After 2 weeks of growth, the cells were fixed and stained with crystal violet, and visible colonies were counted according to the cell numbers in each colony.

### In vivo tumor growth

Nude mice were purchased from the SPF Laboratory Animal Center at Dalian Medical University. All animals were used in accordance with institutional guidelines, and the current experiments were approved by the Animal Care and Use Committee. Bel/Fu cells (1×10^7^/100 μl) were resuspended in PBS and inoculated subcutaneously into 20 6-week-old nude mice. Two weeks later, when tumor diameters reached 4 mm to 5 mm, the mice were randomly divided into 4 groups (n=5/group): 30 mg/kg 5-Fu for the 5-Fu group (5-Fu), 2 mg/kg metformin for the metformin group (Met), 30 mg/kg 5-Fu and 2 mg/kg metformin for the combination treatment group (Com), and PBS alone for the control group (NC). These agents were intraperitoneally injected three times per week for six weeks. The tumors were measured weekly, and tumor volume was calculated according to the formula length × width^2^/2. The mice were terminated six weeks after inoculation, and the weights of the tumors in each mouse were measured.

### Flow cytometric analysis

Cells were seeded into 6-well plates (2×10^5^ cells/well). For cell cycle analysis, the cells were harvested and resuspended in 2 ml of ice-cold 70% ethanol at 4°C overnight. The fixed cells were centrifuged and washed with PBS. After incubation with 100 μl of RNase A (10 μg/ml) for 30 min at 37°C, the cells were resuspended in 400 μl of PI (50 μg/ml) and placed in the dark at room temperature for 30 min. For apoptosis analysis, the cells were digested and resuspended in binding buffer to prepare single cell suspensions. Then, the cells were stained using the Annexin V-FITC reaction reagent (5 μl of Annexin V-FITC, 5 μl of propidium iodide) and incubated in the dark at room temperature for 30 min. The stained cells were analyzed using an Accuri C6 flow cytometer.

### TUNEL assay

Evidence of DNA strand breaks was detected using the transferase-mediated dUTP nick-end labeling (TUNEL) assay. The cells were cultured on coverslips for at least 24 h for attachment and then incubated with various treatments at 37°C for 48 h. TUNEL assay was conducted according to the manufacturer's instructions. Fluorescence images were photographed at × 200 magnification under a fluorescence microscope.

### Western blotting

The cells were lysed in RIPA buffer on ice supplemented with 1 mM PMFS and 1 mM phosphatase inhibitor cocktail to obtain total cellular protein. Protein concentrations were determined using a BCA protein assay kit. The protein samples (30 μg) were mixed with loading buffer, separated by 6% or 10% SDS-PAGE and transferred onto PVDF membranes. The membranes were then blocked and incubated with the primary and secondary antibodies. Then, the bands were visualized by chemiluminescence.

### Statistical analysis

SPSS 17.0 statistical software was used for statistical analysis. All experiments were repeated three times. Values were presented as the mean ± standard deviation. Student's t-test, one-way ANOVA and χ^2^ analysis were performed to analyze variance. Survival analysis was conducted using the Kaplan-Meier method, and the comparison of survival curves between groups was done with the log-rank test. A *P*-value < 0.05 represented statistical significance.

## SUPPLEMENTARY FIGURE


