# Supplementary material for: Metformin mediates resensitivity to 5-fluorouracil in hepatocellular carcinoma via the suppression of YAP
Source: Oncotarget. 2016 Jun 15;7(29):46230–41. doi: 10.18632/oncotarget.10079 (PMC5216793; doi:10.18632/oncotarget.10079)
Supplement: Supplementary file 1 [file oncotarget-07-46230-s001.pdf]

Metformin mediates resensitivity to 5-fluorouracil in hepatocellular carcinoma via the suppression of YAP

SUPPLEMENTARY FIGURE

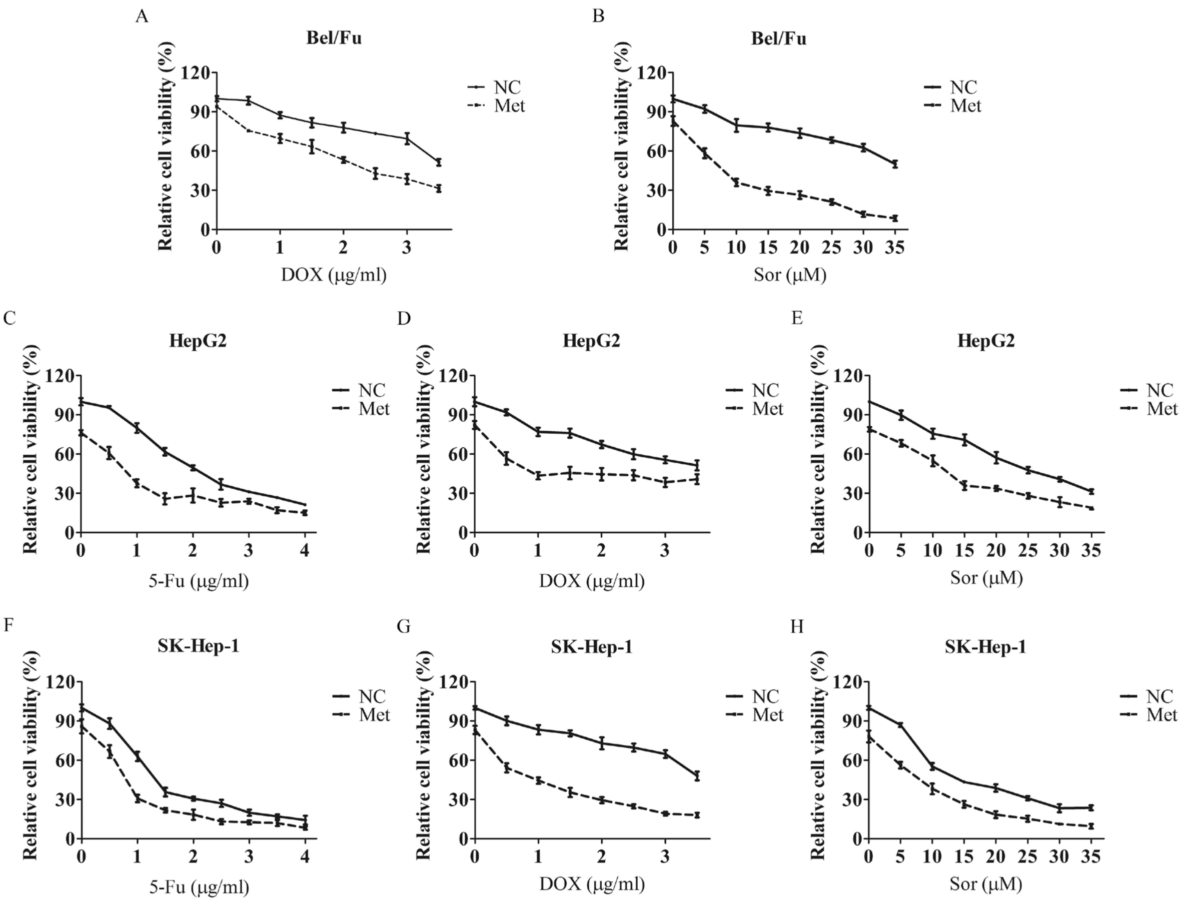

Supplementary Figure S1: Metformin increased sensitivity to chemotherapeutic agents in HCC cells. CCK-8 assay was employed for proliferation analysis 48 h after different treatment in HCC cells. **A, D** and **G.** Comparation of Bel/Fu, HepG2 and SK-Hep-1 cells between DOX and DOX + Met treatment. **B, E** and **H.** Comparation of Bel/Fu, HepG2 and SK-Hep-1 cells between Sor and Sor + Met treatment. **C, F.** Comparation of HepG2 and SK-Hep-1 cells between 5-Fu and 5-Fu + Met treatment.
